# Potential enhancement of post-stroke angiogenic response by targeting the oligomeric aggregation of p53 protein

**DOI:** 10.3389/fncel.2023.1193362

**Published:** 2023-07-18

**Authors:** Hoi Hei Tam, Dongxing Zhu, Samuel Sze King Ho, Heng Wai Vong, Vincent Kam Wai Wong, Simon Wing-Fai Mok, Io Nam Wong

**Affiliations:** ^1^Faculty of Medicine, Macau University of Science and Technology, Macau, Macau SAR, China; ^2^Guangdong Key Laboratory of Vascular Diseases, State Key Laboratory of Respiratory Disease, The Second Affiliated Hospital, Guangzhou Institute of Cardiovascular Disease, Guangzhou Medical University, Guangzhou, Guangdong, China; ^3^Dr. Neher’s Biophysics Laboratory for Innovative Drug Discovery, State Key Laboratory of Quality Research in Chinese Medicine, Macau University of Science and Technology, Macau, Macau SAR, China

**Keywords:** p53, protein aggregation, post-stroke recovery, angiogenesis, CypD

## Abstract

Tumor suppressor gene p53 and its aggregate have been found to be involved in many angiogenesis-related pathways. We explored the possible p53 aggregation formation mechanisms commonly occur after ischemic stroke, such as hypoxia and the presence of reactive oxygen species (ROS). The angiogenic pathways involving p53 mainly occur in nucleus or cytoplasm, with one exception that occurs in mitochondria. Considering the high mitochondrial density in brain and endothelial cells, we proposed that the cyclophilin D (CypD)-dependent vascular endothelial cell (VECs) necrosis pathway occurring in the mitochondria is one of the major factors that affects angiogenesis. Hence, targeting p53 aggregation, a key intermediate in the pathway, could be an alternative therapeutic target for post-stroke management.

## 1. Introduction

Stroke is one of the major health burdens across the world population, representing the leading cause of death worldwide (Allen and Bayraktutan, 2008). Amongst the diversity of strokes as clinically presented, the ischemic type accounts for 85% of all strokes (Orlowski et al., 2011) and occupies around half of the total stroke mortality (Feigin et al., 2021). Although the number of deaths from stroke is decreasing over recent years, permanent impairment of physical abilities remains a main cause of disability [69% of all years lived with disability (YLDs)] (Pacheco-Barrios et al., 2022), particularly affecting the professionally active population (Guzik and Bushnell, 2017). Accordingly, post-stroke recovery aimed to regain patient independence is a significant therapeutic concern, which is highly associated with the cranial milieu during stroke development (subclinical atherosclerosis) and after the ischemic/reperfusion (I/R) process (Qin et al., 2022).

Angiogenesis involving increased vascularization surrounding the infarct areas is observed after stroke (Roll and Faissner, 2014). Such regeneration process, which only took several days for the formation of fully functional new blood vessels (Reitmeir et al., 2012), can stimulate other endogenous mechanisms, including neurogenesis and synaptogenesis, via the restoration of oxygen and nutrients supply (Ergul et al., 2012). Therefore, therapeutically targeting the cerebral vasculature by the facilitation of angiogenesis has been suggested as a promising strategy for stroke recovery (Paro et al., 2022). Together with the fact that the effect of thrombolysis (the only therapy for ischemic stroke) is limited by a short therapeutic window after the infarct (Adams et al., 1996), clinical procedures aimed to enhance the angiogenic responses become especially important. Of note, the tumor suppressor protein p53 is highly associated with the inhibition of angiogenesis as illustrated in many different cancerous diseases (Teodoro et al., 2007). Similar p53-induced inhibitory effects have also been found in stroke mediated by the hypoxia-inducible factor 1α (HIF-1α) and vascular endothelial growth factors (VEGFs) pathways (Pfaff et al., 2018).

Accordingly, this review article particularly focused on the aftermath of ischemic stroke resulting from thrombus formation in the major cerebral blood vessels. The different angiogenesis pathways which are crucial for reperfusion and recovery post ischemic stroke were first reviewed. The potential role of p53 aggregate in the pathological progression of stroke was then suggested and its possible therapeutic effects on improving post-stroke outcomes were discussed (Table 1).

**TABLE 1:** Table summary of the mini-review.

Possible effects of WT p53 aggregates on angiogenesis			
Pathways involving WT p53	Cellular model	Subcellular location	Responses upon p53 aggregate formation
Upregulation of BAI 1	HUVEC	Cytoplasm	Angiogenic
Inhibition of bFGF	Tumor cells both *in vitro* and in mouse model	Nucleus	Angiogenic
Binds VEGF promotor	Retinoblastoma cells	Nucleus/cytoplasm	Angiogenic
Binds HIF-1α	HCT116/MEFs	Nucleus/cytoplasm	Angiogenic
Inhibition of VEGF (Rb-p21 dependent)	HCT116/MEFs	Nucleus	Angiogenic
Direct inhibition of NF-κB	NIH3T3 (mouse)/P19 (hamster)	Nucleus	Angiogenic
Upregulate Wnt-11	MCF10A (human)	Extracellular	Angiogenic
CypD catalyzed p53 aggregation*	HEK293	Mitochondria	Anti-angiogenic
*This is proposed to be the major pathway in stroke patients and outweighs other pathways listed above.			
**Determinants of WT p53 misfolding/Aggregation**			
Intrinsic factor		Extrinsic factor	
DBD instability		Temperature	
High p53 level		pH	
Mutations (rarely reported in stroke)		Oxidative and inflammatory stress	
**Other protein aggregate observed in stroke brain**			
SUMO			
RNABPs			
PSF			
NONO			

## 2. Pathogenesis of ischemic stroke

Ischemic stroke is defined as an abrupt interruption of cerebral blood flow predominantly caused by atherosclerotic thrombosis, leading to persistent blockage of oxygen and essential nutrients delivery to the brain (Radak et al., 2017). The initiation of cerebral oxygen deprivation can even begin as early as the developmental stage of atherosclerosis when the intimal becomes increasingly thickened as a result of vascular plaques buildup (Nakagawa and Nakashima, 2018). Therefore, the stroke-associated hypoxic environment is covering the period since thrombus formation, then all the way to plaque rupture, and eventually after the occurrence of ischemic stroke (Ferdinand and Roffe, 2016). Direct consequence of these pathological events is the depletion of energy stores in cerebral cells, mainly neurons, leading to pan-necrosis of the affected brain tissues within minutes when vital metabolites supply becomes significantly low (Ferdinand and Roffe, 2016). Two mechanisms are responsible for such cell death process: (1) The intrinsic pathway which relies on elevated cytosolic level of calcium ions and the downstream mitochondrial disruption; (2) The extrinsic pathway which involves the binding of death signaling ligands to corresponding receptors on the cell surface (Broughton et al., 2009). The resulting stressed or dying neuronal cells also creates an oxidative and inflammatory milieu by the release of damage-associated molecular patterns (DAMPs) (Iadecola et al., 2020). Of note, endothelial cells constituting the vasculature draining the brain are one of the active participants implicating the progress of stroke, many detrimental molecular and cellular responses were associated with the stroke-induced cerebral endothelial damage (Andjelkovic et al., 2019), which suggests the importance of restoring proper endothelial functioning in post-stroke recovery (Xiang et al., 2018).

## 3. Targeting angiogenesis for post-stroke therapy

Since hypoxia commonly occurs following stroke and represents the major obstacle barricading post-stroke recovery, targeting the enhancement of angiogenesis has become an emerging therapeutic strategy (Paro et al., 2022). Although angiogenesis is an adaptive mechanism developed in the brain after stroke (Zhu et al., 2021), the formation of new blood vessels may be insufficient to support long-term functional recovery involving its capacity to regulate neurogenesis (Ma et al., 2021). As such, to understand the molecular network underpinning angiogenesis is of great importance for exploring the therapeutic potential of angiogenesis enhancement for post-stroke therapy.

Owing to the critical role of VEGFs in angiogenesis (Shibuya, 2011), members of the VEGFs family have emerged as one of the most widely examined therapeutic targets for post-stroke recovery. For example, intracranial administration of VEGFs improves motor coordination and increases vascular density of an ischemic rat model created by cerebral artery occlusion (Sun Y. et al., 2003). Also, knockout of the placental growth factor (PlGF) gene is closely related to the delaying of hypoxia-induced brain angiogenesis as demonstrated in a mouse model (Freitas-Andrade et al., 2012). However, the tactics of stimulating blood vessels regrowth by the elevation of VEGFs level could be harmful without optimizing the applied dosage, since study showed that the resumed blood supply to ischemic area is “stolen” from other non-ischemic area, which could create further damages to the originally unaffected area when VEGFs is overly dosed (Wang, 2004). Effort has been made to overcome such challenge for drug delivery by using hydrogel for stroke treatment due to its site-specificity and modifiable drug release rate (Wilson et al., 2020; Bai et al., 2022; Lei et al., 2022). However, the efficacy of using hydrogel as carrier may be limited by the level of other angiogenic factors, such as angiopoietins and platelet-derived growth factor (PDGF), which are also essential for VEGFs-mediated vascular remodeling, resulting in unsatisfactory angiogenic effect even under optimized amount of VEGF (Lindahl et al., 1999; Fagiani and Christofori, 2013). Although other strategies involving the subtle manipulations of hypoxia-induced angiogenesis after stroke have been suggested (Rascón-Ramírez et al., 2021), these approaches also run into the problem of over-angiogenesis and the formation of dysfunctional tissue architecture.

As inspired by the central idea of optimizing vascular growth to prevent dysfunctional vasculature formation, it is tempted to question if the promotion of post-stroke survival of VECs could provide enough time to facilitate the angiogenesis spontaneously induced after the occurrence of stroke and the later recovery process thereof.

## 4. The regulatory role of p53 in angiogenesis relating to stroke

**FIGURE 1:**
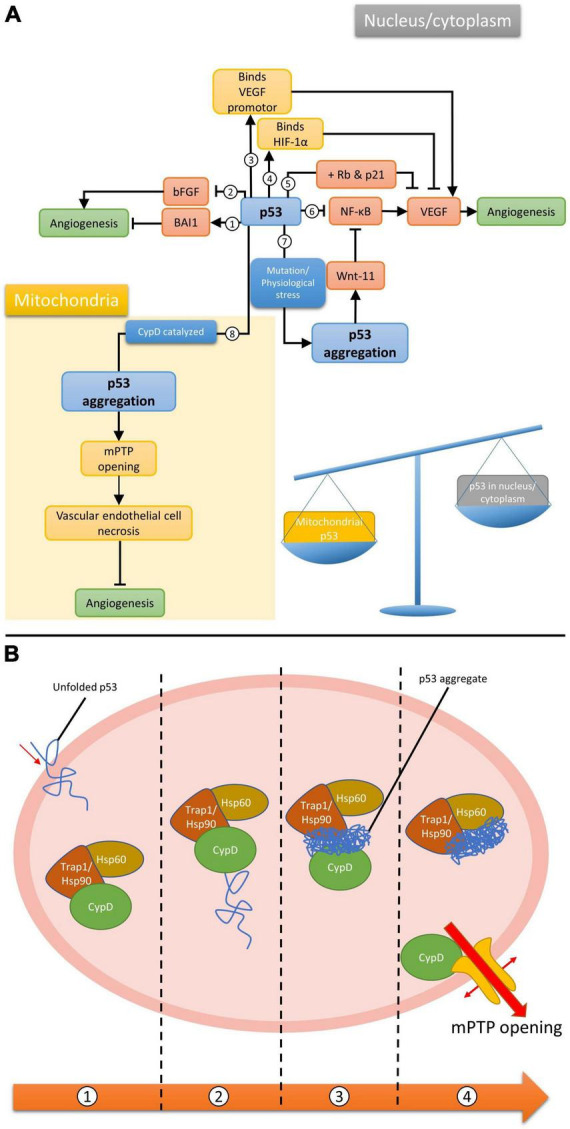
**(A)** Different p53-dependent angiogenesis pathways. Wild-type p53 (1) upregulates BAI1, (2) inhibits bFGF, (3) binds to VEGF promotor synergistically with HIF-1α to upregulate VEGF, (4) binds to HIF-1α to inhibit VEGF, (5) inhibit VEGF when both Rb and p21 are present, (6) inhibits NF-κB, which promotes VEGF expression. It can also form aggregation via mutation/physiological stresses in the nucleus, (7) promoting wnt-11 which inhibit NF-κB, or (8) via CypD catalysis in mitochondria; **(B)** CypD catalyzed mPTP opening pathway. (1) Unfolded p53 enters the mitochondrial matrix. (2) p53 binds to and activates CypD as an isomerase. (3) CypD isomerized p53 and promote p53 amyloid formation. (4) p53 aggregate binds to chaperones and frees up CypD for mPTP opening.

Wild-type (WT) p53 is well known for its inhibitory effect on the angiogenesis associated with tumorigenesis (Teodoro et al., 2007), which can be mediated by several molecular mechanisms (Figure 1A). For example, p53 downregulates endogenous VEGFs expression by binding to its transcription factor Sp1 (Pal et al., 2001) or inhibition of NF-κB (Ko et al., 2006). On the other hand, the amount of the oxygen sensor HIF-1α, which is responsible for the activation of VEGFs, can be reduced by p53-mediated ubiquitination under hypoxic microenvironment (Ravi et al., 2000). However, p53 and HIF-1α has also been demonstrated to upregulate VEGF expression through synergistically binding to the VEGF promoter in acute hypoxia (Farhang Ghahremani et al., 2013). Under persistent deprivation of oxygen, opposing effect has been observed: Downregulation of VEGF can be achieved indirectly by p53 via the retinoblastoma (Rb) and p21-dependent pathway (Farhang Ghahremani et al., 2013). Other pro-/anti-angiogenic factors, including basic fibroblast growth factor (bFGF) and brain-specific anti-angiogenic inhibitor 1 (BAI1) have also been reported to supress angiogenesis in a p53-dependent manner (Ueba et al., 1994; Nishimori et al., 1997; Sherif et al., 2001). Apart from the above-mentioned pathways, those involving the oligomeric aggregation of p53 protein are of great interest as the inhibition of p53 aggregate may indirectly promote angiogenesis by preventing VECs necrosis (Lebedev et al., 2016). Intriguingly, WT p53 protein is significantly increased in the ischemic stroke brain (Luo et al., 2009; Almeida et al., 2021), therefore, its anti-angiogenic capacity as observed in neoplastic tissues may also happened in the brain after ischemic stroke. Such notion is supported by the viability of pifithrin-α, an inhibitor of p53, on promoting regenerative repair and angiogenesis post ischemic stroke (Zhang et al., 2016).

## 5. The potent implication of p53 aggregate in angiogenic response

Earlier research works revealed that aggregation of proteins, including ubiquitin and small ubiquitin-like modifier (SUMO), are involved in neuronal injuries induced by ischemic stroke (Hayashi et al., 1992; Hu et al., 2001; Zhang et al., 2006; Yang et al., 2008), and since then the number of aggregating proteins identified to be associated with ischemia/reperfusion kept increasing. According to recent findings, the RNA-binding proteins (RNABPs) is thus far the largest group of protein demonstrating aggregation tendency found in the ischemic brain (Kahl et al., 2018). It is worth noting that, many of these proteins such as ubiquitin, SUMO, and RNABPs like PTB-associated splicing factor (PSF) and Non-POU Domain Containing Octamer Binding (NONO) are genetically intact (Hu et al., 2001; Yang et al., 2008; Kahl et al., 2018), suggesting that brain microenvironment during the progression of stroke favors the protein aggregation process. Indeed, protein aggregates appeared to modulate the pathogenesis of cerebral ischemia, therefore, their formation may represent a novel mechanism driving cell dysfunction and death after stroke (Wu and Du, 2021).

The WT p53 protein has a high tendency to aggregate naturally due to the constituting unstable DNA binding domain (DBD) and an amyloidogenic sequence (Cino et al., 2016). In fact, WT p53 aggregation can even occur under physiological conditions (Julian et al., 2022). Together with the significantly upregulated cerebral level of p53 after stroke, it is reasonable to postulate that p53 is also capable of forming aggregate in the stroke brain. However, findings related to p53 have been focusing on neurogenesis due to its important role in neuronal cell death (Balaganapathy et al., 2018; Xu et al., 2023). Although research studies relating p53 aggregates to angiogenesis or VECs are yet limited, such phenomenon would most likely happen when integrating associated findings in other cellular and animal models. For example, the gain of function upon the formation of amyloidal p53 upregulates Wnt-11, a protein that promotes anti-inflammatory effect, in MCF10A human mammary epithelial cell model (Sengupta et al., 2022). Increased expression of Wnt-11 was also observed to inhibit the NF-κB pathway in cardiac progenitor cells (Bisson et al., 2015), NIH3T3 cells, P19 embryonic carcinoma cells (Maye et al., 2004) and Chinese hamster ovary cells (Du and Geller, 2010), suggesting the possibility of controlling VEGF expression hence angiogenesis by p53 aggregates (Figure 1A, pathway 7).

A different model for p53-dependent angiogenesis regulation was proposed by Lebedev et al. (2016) suggesting the role of p53 and how the formation of its aggregation could lead to cell necrosis. Partial unfolded p53 induced by various stresses is able to translocate into the mitochondrial matrix, where it will activate cyclophilin D (CypD) toward an isomerase by forming p53-CypD complex. Activated CypD will then catalyze the cis/trans prolyl isomerization of p53, leading to irreversible formation of p53 aggregation. The aggregated p53 can chelate to the molecular chaperones in the mitochondria and frees up CypD. This allows the isolated CypD to bind to the mitochondrial permeability transition pore (mPTP), inducing the opening of the pore, eventually leading to necrotic cell death. Research has shown the importance of CypD and mPTP in regulating endothelial cells and hence angiogenesis (Marcu et al., 2015). Given that many factors during and after stroke would destabilize WT p53 and lead to the formation of aggregation, we propose these pathways could play an important role in managing post-stroke angiogenesis, thus the recovery from acute stroke.

## 6. Determinants of p53 unfolding after stroke for p53 aggregation

In general, protein aggregation begins with the unfolding or misfolding of a particular protein from its native conformation, which exposes the hydrophobic core for intramolecular interactions with other unfolded monomers (Wang and Roberts, 2018). The correct folding of protein is usually disturbed by the loss of genetic integrity, such as point mutation, which alters the intramolecular forces that are responsible for maintaining protein structures, leading to a number of human diseases. In the case of p53, the R248Q mutation in the DBD is highly associated with the increased rate of aggregate formation. The accumulation of such aggregates was observed in different cancers such as neuroblastoma, retinoblastoma, breast, and colorectal cancers (Ano Bom et al., 2012).

**FIGURE 2:**
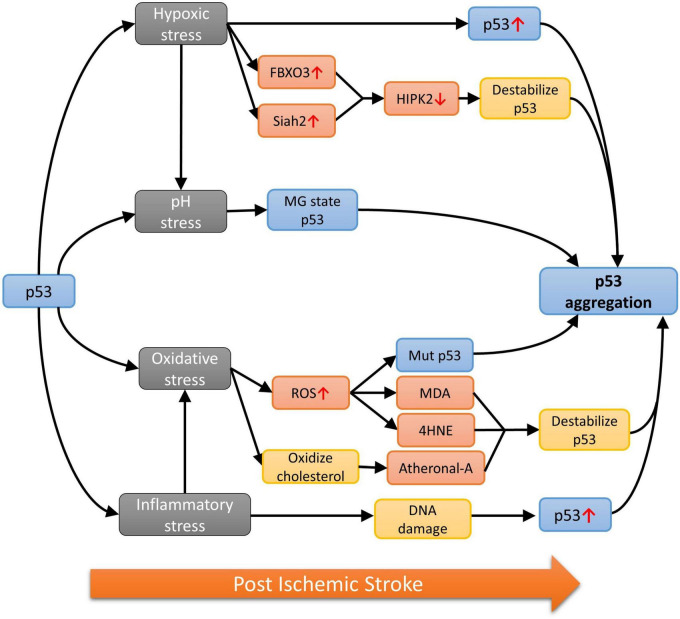
The p53 aggregation pathways under different stresses.

In contrast, findings demonstrating the presence of stroke-specific or aggregation-prone p53 mutations are still scarce, which implies the involvement of WT p53 and their self-aggregation during stroke progression. It is well-known that the DBD of WT p53 is an unstable structure, which has a high tendency for self-aggregation, especially when the backbone hydrogen bonds are poorly stabilized and exposes the amyloidogenic sequence (Cino et al., 2016). It is worth noting that the stroke-associated cranial milieu is capable of accelerating the destabilization of p53. These factors that could induce the accumulation, misfolding, oligomerization or even toxic amyloidogenic aggregation of p53 in stroke, include the oxygen level, pH change, generation of free radicals and immune status in the affected brain via a complex network of molecular pathways (Figure 2).

### 6.1. Hypoxia

Oxygen deprivation in the brain is commonly found after stroke featuring the clinical presentation of irreversible brain damages (Ferdinand and Roffe, 2016). Low oxygen level also induces p53 accumulation (Koumenis et al., 2001) and skews the cellular environment to favor the unfolding of p53 via different pathways. For example, the homeodomain-interacting protein kinase 2 (HIPK2) is the main mediator for destabilizing the native p53 under hypoxia by phosphorylating Ser46 in p53 (Puca et al., 2008). The HIPK2 level is in turn negatively controlled by the E3 ubiquitin ligase F-Box Protein 3 (FBXO3) and Siah2 in an oxygen-dependent manner. As demonstrated in cellular and animal models subjected to hypoxic changes, expression of the two ubiquitin ligases is upregulated and interacts with HIPK2 to promote the proteasomal degradation of the protein (Calzado et al., 2009; Gao et al., 2022). Hence, HIPK2 level is expected to be suppressed by increased rate of degradation due to the large amount of FBXO3 and Siah2 post ischemic stroke.

### 6.2. pH stress

The hypoxia environment of ischemic stroke also favors anaerobic glycolysis, resulting in the build-up of lactic acid, which can lower the pH to as low as 6.0 in the ischemic core (Tóth et al., 2020). Additionally, the increase in partial pressure of CO_2_ (pCO_2_) is also shown to contribute to pH drop after ischemic stroke (Hanwehr et al., 1986). Conformation of WT p53 has also been shown to be strongly correlated with pH changes and acquires a partially unfolded molten-globule (MG) state, a more aggregation-prone structure when compared with the native state (Pedrote et al., 2018), at pH 5.0 (Ano Bom et al., 2010). As demonstrated in the ^1^H-^15^N HSQC performed at pH 5.0 and 7.2, the alteration of the p53 structure caused by pH change is not limited to one domain but the entire protein (Tóth et al., 2020). It is worth noting that the drop from physiologically normal pH to pH 5.0 is a dramatic change that is unlikely to occur in stroke and p53 might not fully adopt the MG state as such. However, finding from another experiment suggested that p53 structure can be opened to expose the hydrophobic core at pH 6.0, which may eventually lead to p53 aggregation in stroke (Pedrote et al., 2018).

### 6.3. Oxidative and inflammatory stress

Owing to the low neuronal antioxidant activity, the stroke brain has a higher tendency than other organs to build-up ROS and reactive nitrogen species (RNS). Free radicals could lead to mutation or oxidation of p53, exposing the cysteine residues, which can attack other cysteine residues on other p53 to form disulfide bonds (Sun X. Z. et al., 2003), eventually forming dimers, oligomers, or even larger aggregates of p53 (Hainaut and Milner, 1993). On the other hand, unfolded p53 has been found to translocate into the mitochondria and binds to CypD from its N-terminal domain, forming an enzyme-substrate like CypD-p53 complex (Zhao et al., 2022). Furthermore, the binding of p53 will activate CypD toward an isomerase which will then cause prolyl isomerization of p53 and lead to aggregation (Vaseva et al., 2012; Lebedev et al., 2016). Different aldehydes will also be formed when subject to oxidative stresses and research had shown that the α, β-unsaturated aldehydes such as malondialdehyde (MDA), 4-hydroxynonenal (4HNE) and 4-hydroxyhexenal, all resulted from free radical attack on fatty acids in the neuronal membrane, are only able to cause p53 aggregation when thioredoxin reductase (TrxR) is present (Cassidy et al., 2006). However, TrxR function had been shown to reduce in the ischemic brain of mouse model (Zhao et al., 2020), thus this might not be the major pathway for aggregation when subject to oxidative stress. In contrast, inflammation-induced oxidation can oxidize cholesterol to form stronger, harder electrophile atheronals which can cause p53 aggregation by interacting with lysine residues on p53 (Nieva et al., 2011). Additionally, neuroinflammatory cytokines such as the tumor necrosis factor α (TNF-α), interleukin 1β and 6 (IL-1β and IL-6) would cause DNA damages in stroke patients, triggering the upregulation of WT p53 as a protective mechanism (Fu et al., 2019; Ibrahim et al., 2020; Abuetabh et al., 2022). The accumulated p53 will also subject to the oxidative stresses, making them more aggregation-prone, hence increasing the chance of forming amyloidal fibril.

### 6.4. Other possible mechanisms

Another factor to note is that ischemic stroke also disrupts the protein quality control system (Thuringer and Garrido, 2019; Wu and Du, 2021) by reducing the stabilization received from the molecular chaperones, thereby increasing the risk of protein aggregation in general (Douglas et al., 2009). Further, the abnormal expression of non-coding RNAs has been hypothesized to be associated with promotion of RNA-binding protein aggregation after stroke (Wu and Du, 2021). Non-coding RNAs such as MEG3, PSTAR and PANDA has been linked to the stability of WT p53 in other cellular models, suggesting their possible role in p53 aggregate formation after stroke (Kotake et al., 2016; Jain, 2020).

## 7. Inhibition of p53 aggregation as potential therapeutic strategy

Since p53 has a high potential in forming aggregation in the stroked brain, together with the fact that WT p53 is highly involved in angiogenic process, it is reasonable to propose that p53 aggregates may be mechanistically involved in the regulation of CypD-dependent necrotic pathway occurring in the mitochondria of VECs. One thing worth noting is that the inhibition of p53 aggregation would lead to further accumulation of WT p53, which suppresses the expression of VEGF in the nucleus. Therefore, inhibition of p53 aggregation would either be: (1) Pro-angiogenic if the CypD/mitochondria pathway is dominant since the suppression of p53 aggregation would prevent CypD from moving freely in the mitochondrial matrix, leading to the inhibition of mPTP opening and preventing the necrotic death of VECs; or (2) anti-angiogenic when the VEGF/nucleus arm is considered, as most p53-dependent angiogenesis pathways in the nucleus that have discussed in this mini-review would result in anti-angiogenic effect when subject to p53 aggregate inhibition. Therefore, the determining factor for the overall effect of targeting p53 aggregate is likely to be the subcellular location of p53 and its aggregates.

In the stroke brain, more unfolded p53 is likely to be translocated into mitochondria rather than the nucleus as the number of mitochondria observed in cerebral VECs is considerably high (Lee et al., 2020). Hence, the protective effect on VECs upon p53 aggregation inhibition via the CypD-dependent pathway is expected to outweigh the other anti-angiogenic pathways in the nucleus and ends up with a net pro-angiogenic effect. In addition, large number of mitochondria in VECs constituting the blood brain barrier (BBB) and neurons is also observed due to the high energy demand of the brain, suggesting the potential neuroprotective and regenerative effect on neurons (Han et al., 2016), as well as the protective effect on BBB (Zhang et al., 2020) when adopting this therapeutic strategy.

On top of the potential therapeutic effect, such strategy also avoids the hurdle of other p53-based therapies by the direct manipulation of p53 level. Hence, the inhibition of p53 aggregate formation or to simulate the refolding of dissembled p53 may be a better alternative to the current therapies. On the other hand, researchers have identified CypD as an important therapeutic target for managing mPTP opening (Waldmeier et al., 2003; Peterson et al., 2022) and the inhibition of which has been shown to rescue mitochondria in bone cells (Gan et al., 2018), damages on the liver (Panel et al., 2019) or brain (Sun et al., 2017) after ischemia/reperfusion injury. All these supports our hypothesis that mitochondria-mediated VECs necrosis plays an important role in I/R injury and inhibiting the pathway would improve the outcome after stroke. However, the possible contribution of CypD to other pathways (Porter and Beutner, 2018) may cause unwanted side effects upon inhibition. Manipulation of the CypD-dependent necrosis by targeting p53 aggregates could avoid this problem, thus reducing the complexity during drug development.

## 8. Outlook

In this review, we proposed a novel post-stroke recovery strategy by suppressing VECs necrosis via the inhibition of p53 aggregation. The advantage of such an approach is to avoid the unwanted adverse effects induced by the direct promotion of angiogenesis. Instead, the new strategy aims to protect VECs from necrosis, extending their survival time to allow sufficient angiogenesis for post-stroke recovery. The currently proposed pathway may provide novel insight to support further investigation on targeting p53 aggregation in post-stroke recovery by using known p53 aggregation inhibitor such as small peptide ReACp53 (Soragni et al., 2016) or small natural compounds (resveratrol and emodin) (Ferraz da Costa et al., 2018; Haque et al., 2018). Also, the use of phage/iPhage (internalizing phage) display and other structure-based drug discovery approaches can be effective in identifying novel therapeutic peptides/drug compounds for preventing p53 aggregation as the new treatment for stroke or post-stroke management (Tosstorff et al., 2019; Zhang et al., 2022).

## Author contributions

INW and SWFM conceived the idea of the manuscript. INW, SWFM, and HHT wrote the original draft for the manuscript. All authors contributed to the manuscript revision and editing and approved the submitted version.
